# Supplementary material for: Cavitand Decorated Silica as a Selective Preconcentrator for BTEX Sensing in Air
Source: Nanomaterials (Basel). 2022 Jun 27;12(13):2204. doi: 10.3390/nano12132204 (PMC9268237; doi:10.3390/nano12132204)
Supplement: Supplementary file 1 [file nanomaterials-12-02204-s001.zip › nanomaterials-1764862-supplementary.pdf]

Supporting Information

Cavitand decorated silica as selective preconcentrator for BTEX sensing in air

Andrea Rozzi[a], Alessandro Pedrini[a], Roberta Pinalli[a], Enrico Cozzani[b], I. Elmi[c], Stefano Zampolli[c], Enrico Dalcanale[a]*

[a] *Department of Chemistry, Life Science and Environmental Sustainability and INSTM UdR Parma, University of Parma Parco Area delle Scienze 17/A and INSTM UdR Parma 43124 Parma, Italy.*

[b] *PROAMBIENTE S.c.r.l., Via P. Gobetti 101, 40129 Bologna, Italy*

[c] *CNR-IMM Bologna, Via P. Gobetti 101, 40129 Bologna, Italy*

Index

1 General experimental methods **2**

2 NMR and MS spectra **5**

3 IR spectra **8**

4 SEM analyses **9**

5 Preconcentrator testing conditions **10**

6 References **11**

1 General experimental methods

Synthesis

Unless stated otherwise, all reactions were carried out under strictly anhydrous conditions under Ar atmosphere. All solvents were dried and distilled using standard procedures. All commercially obtained reagents were used as received unless otherwise specified. Silica column chromatography was performed using silica gel (Fluka 230 – 400 mesh). The precursor QxCav with four ω-decenyl feet was prepared following a published procedure [1]. Monosilylated QxCav was synthetized adapting published procedures [2,3].

NMR spectroscopy

NMR spectra were collected on Bruker Avance 400 (400 MHz) spectrometer at 25 °C. ^{1}H and ^{13}C NMR chemical shifts (δ) are given in part per million (ppm) and calibrated to either residual solvent signal. NMR data are reported in the following format: chemical shift (multiplicity (s = singlet, d = doublet, t = triplet, q = quartet, dd = doublet of doublets, m = multiplet), coupling constants (Hz), integration). The spectra are shown in Figures S2-S5.

ATR-IR spectroscopy

Infrared absorption spectroscopy analyses were performed with a Perkin Elmer FT-IR Spectrum Two instrument using powder samples. The background was subtracted from every spectrum recorded.

TGA analysis

TGA analysis were performed on a Perkin Elmer TGA 8000. Heating run: 25 to 900°C at 10°C/min, in air.

Mass spectrometry

High-resolution MALDI-TOF was performed using an AB SCIEX MALDI TOF-TOF 4800 Plus (matrix: α-cyano-4-hydroxycinnamic acid). The spectrum is shown in Figure S6.

Scanning Electron Microscopy

SEM images were collected with a Zeiss SUPRA 40, at EHT of 2.0 kV. Prior to SEM analysis, the samples were sputtered onto a carbon tape. EHT

MEMS devices of the mini-GC system

The mini-GC system used for the comparative cavitand characterizations relies on two microfluidic MEMS devices implementing the two main analytical functions: purge&trap pre-concentration and gas-chromatographic (GC) separation.

The pre-concentration MEMS cartridge is a 25x14x1.5 mm^3 silicon/borosilicate stack containing a 90 mm^3 reservoir for the cavitand mesh etched into the silicon wafer by deep reactive ion etching. The geometry of the reservoir consists of 8 parallel channels, 1 mm wide and 17 mm long, with two common sections connecting them all to the inlet and outlet holes drilled in the borosilicate wafer (Figure S1 left, blue).

The GC MEMS is a 25x25x1.5 mm^3 silicon/borosilicate stack integrating a 50 cm long double spiral channel, which is 0.8 mm wide and 0.65 mm deep, acting as packed GC column (Figure S1, right, red).

Both MEMS devices integrate Platinum thin film metallization, the silicon wafer as heater and temperature sensor. The small thermal mass of the MEMS device enables very rapid temperature cycling with heating rates up to 10 °C/s for the preconcetrator MEMS, as necessary for an efficient purge&trap injection.

Small MEMS filters at the outlet port of the MEMS devices are used to retain the mesh during the filling procedure, where vacuum is applied at the filter side pull the mesh into the reservoir. In case of the pre-concentrator, the mesh consists of the cavitand material, either in pure form or grafted on silica gel. In case of the GC separation column, the mesh is a Carbograph™ based packed stationary phase.

The following figure shows the geometries of the two MEMS devices, while the detailed clean-room fabrication procedure is reported elsewhere [4].

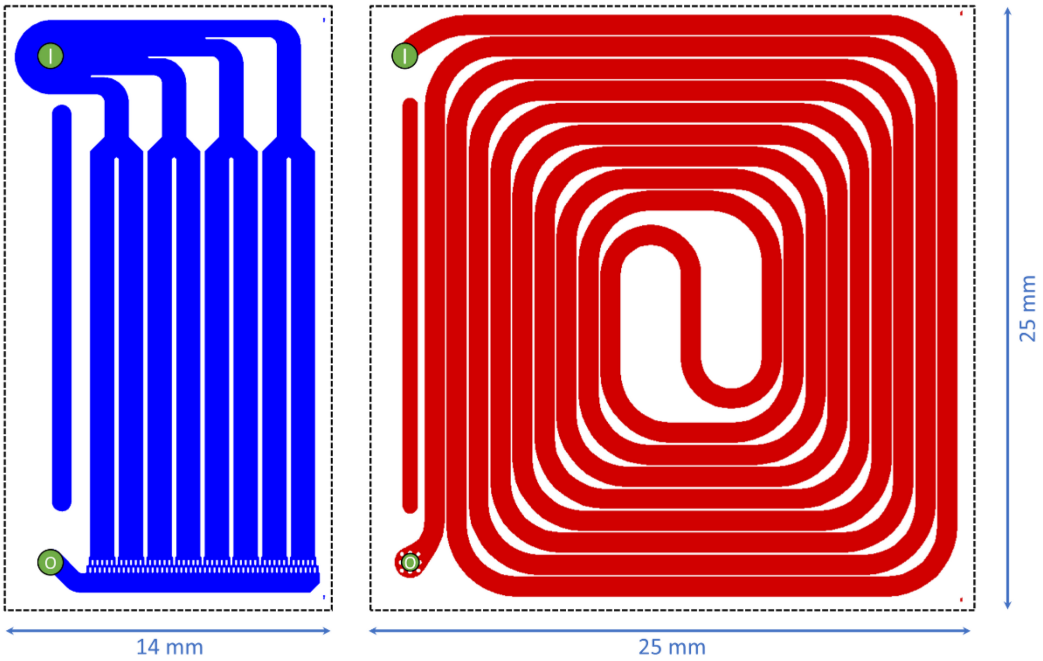

Figure S1. Geometries of the channels fabricated by deep reactive ion etching for the pre-concentrator (left, blue) and GC separation column (right, red) MEMS. The green circles represent the inlet (I) and outlet (O) holes laser-drilled through the borosilicate wafer.

2 NMR and MS spectra

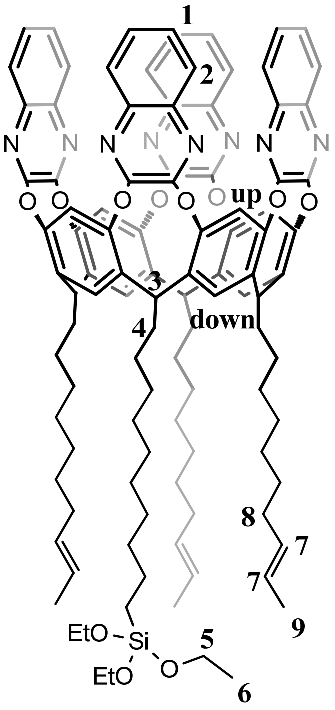

^{1}H NMR (400 MHz, $CDCl_3$) δ = 8.17 (s, 4H, H up), 7.82 (dd, J = 6.4, 3.4 Hz, 8H, H1), 7.50 (dd, J = 6.3, 3.5 Hz, 8H, H2), 7.25 (s, 4H, H down), 5.58 (t, J = 8.0 Hz, 4H, H3), 5.52 – 5.40 (m, 6H, H7), 3.85 (q, J = 7.0 Hz, 6H, H5), 2.28 (m, 8H, H4), 2.13 – 1.97 (m, 6H, H8), 1.68 (m, 9H, H9), 1.55 – 1.36 (m, 48H, -CH_2-), 1.26 (t, J = 7.0 Hz, 9H, H6).

^{13}C NMR (101 MHz, $CDCl_3$) δ = 152.5, 152.4, 139.6, 135.7, 131.6, 129.0, 129.0, 128.2, 127.7, 124.7, 123.4, 118.7, 58.3, 34.3, 33.3, 32.7, 32.4, 29.7, 29.6, 29.3, 29.2, 28.0, 28.0, 27.0, 25.6, 22.9, 22.8, 21.5, 18.3, 18.0, 14.2, 14.1, 12.8, 10.4.

MALDI-TOF: m\z calculated for $[M+H]^+$ $C_{106}H_{121}N_8O_{11}Si$ = 1709.8878, found = 1710.8937, minor peaks: 1546.8072 (no silyl group), 1874.9801 (two silyl group).

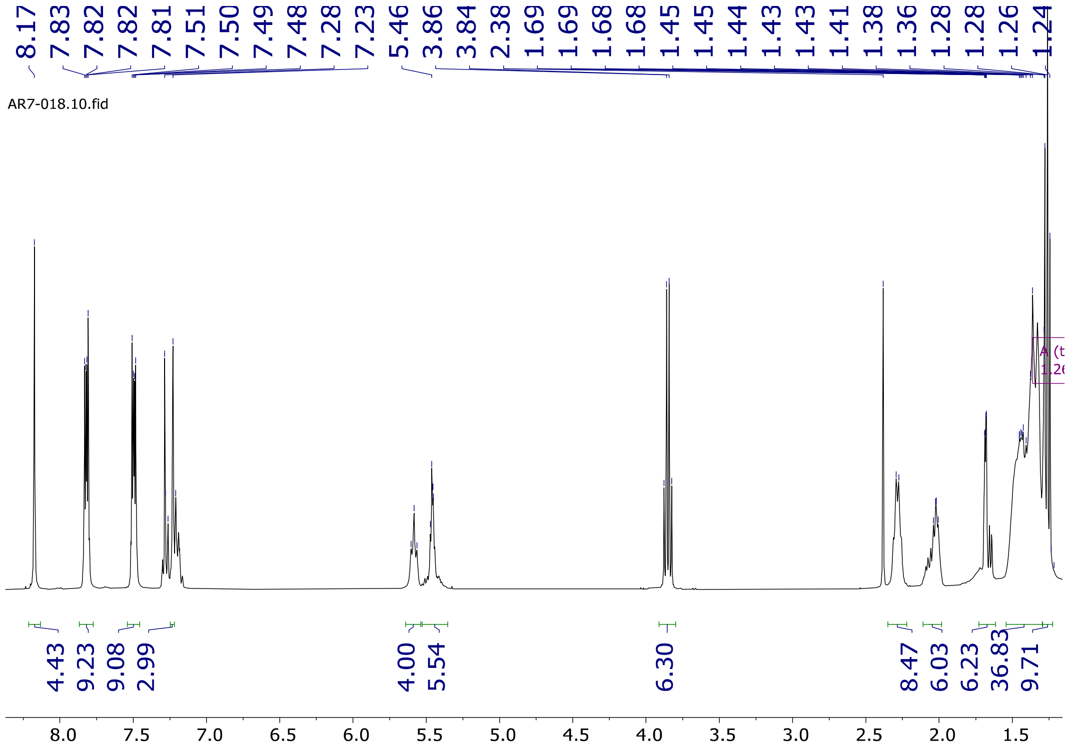

Figure S2. ^{1}H NMR (400 MHz, $CDCl_3$) spectrum of monosilylated QxCav.

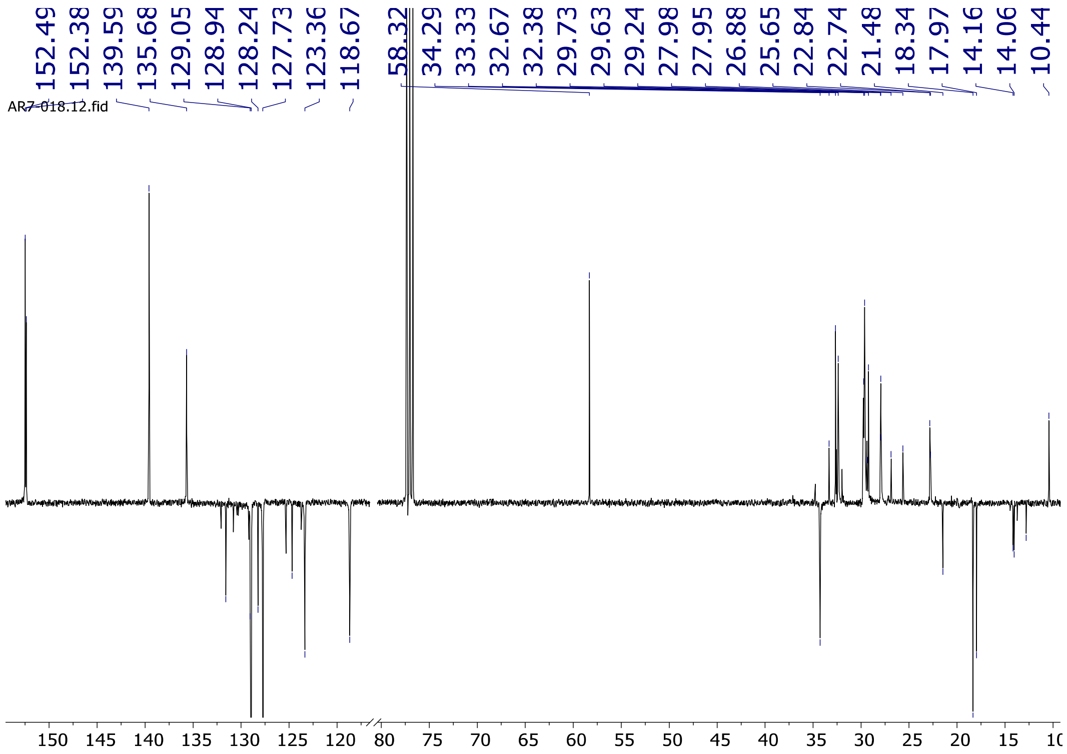

Figure S3. ^{13}C NMR (101 MHz, $CDCl_3$) spectrum of monosilylated QxCav.

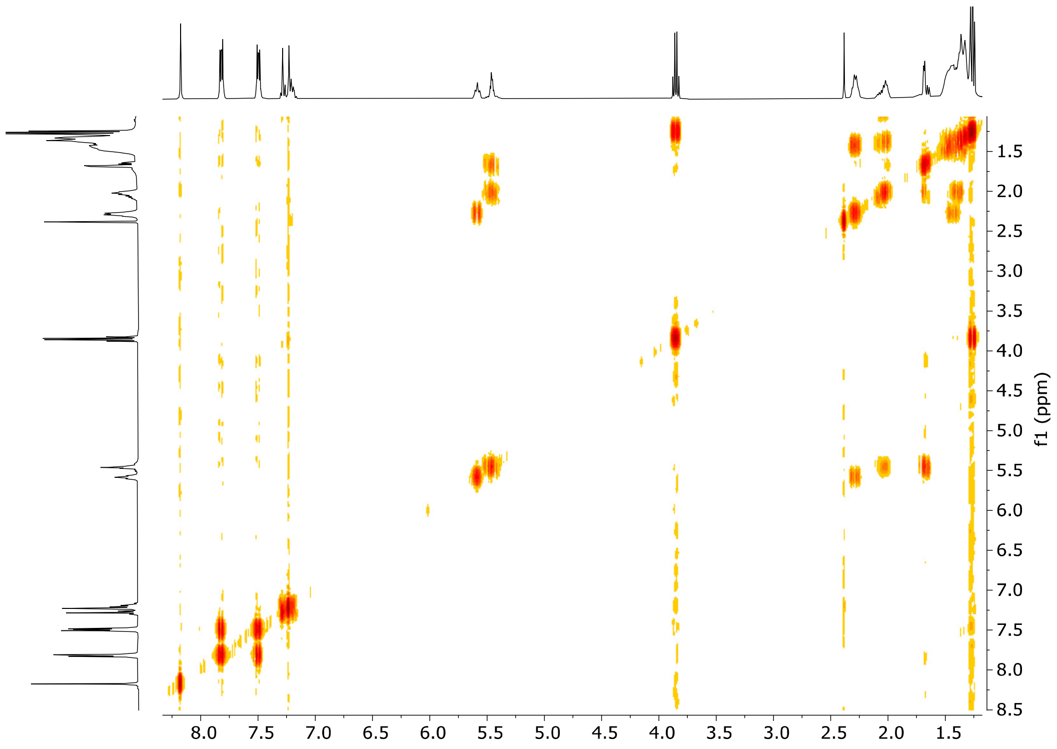

Figure S4. ^{1}H-^{1}H COSY spectrum of monosilylated QxCav.

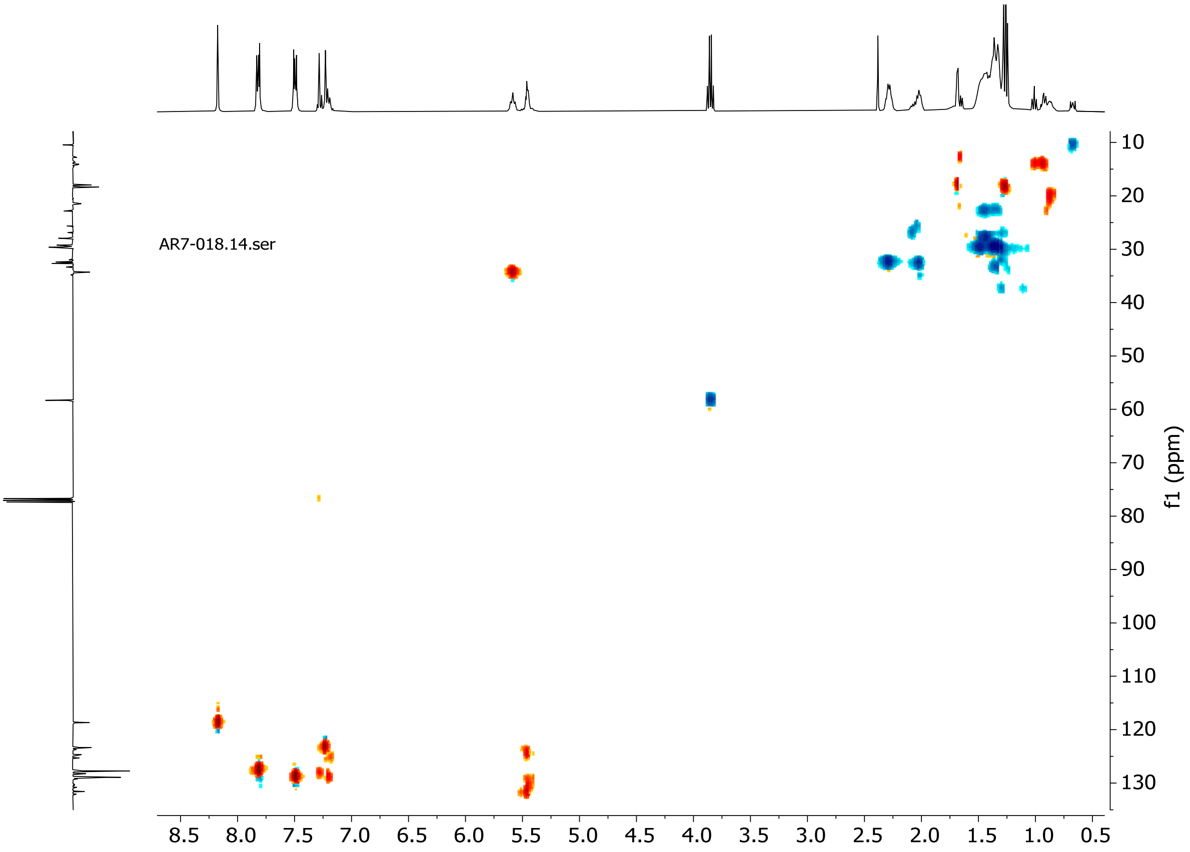

Figure S5. ^{1}H-^{13}C HSQC spectrum of monosilylated QxCav.

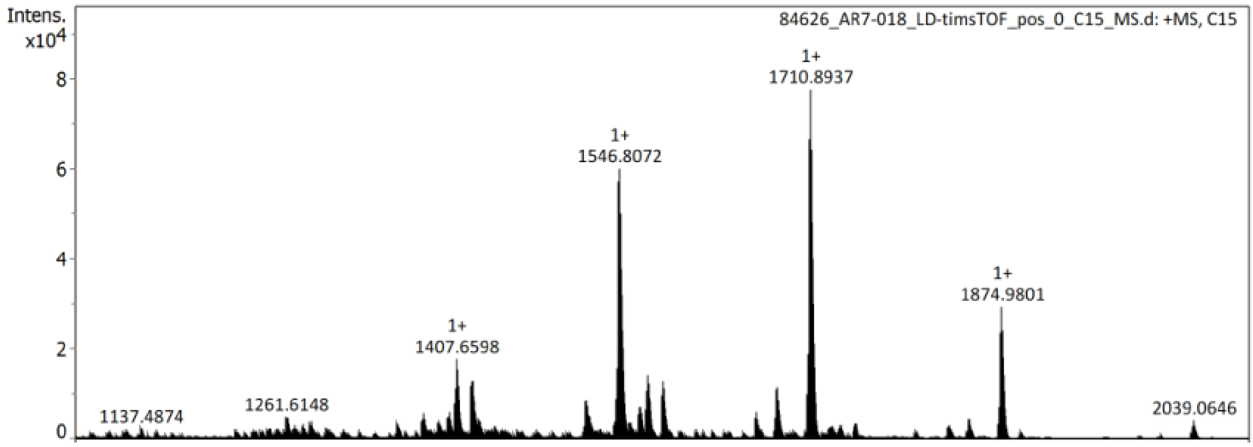

Figure S6. MALDI-TOF spectrum of monosilylated QxCav.

3 IR spectra

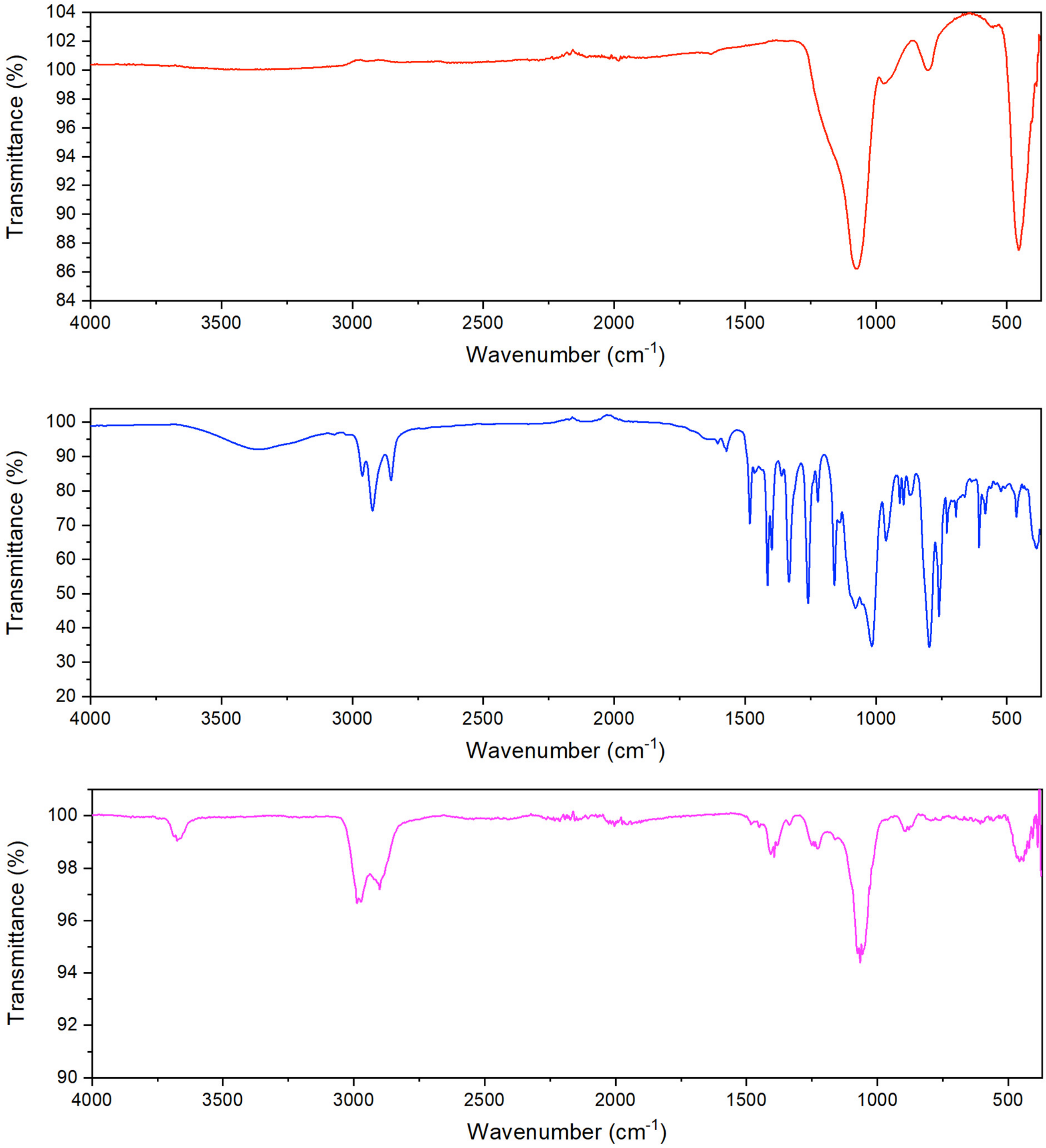

Figure S7. IR spectra of silica (red), monosilylated QxCav (blue) and QxCav@SiO_2 (magenta).

4 SEM analyses

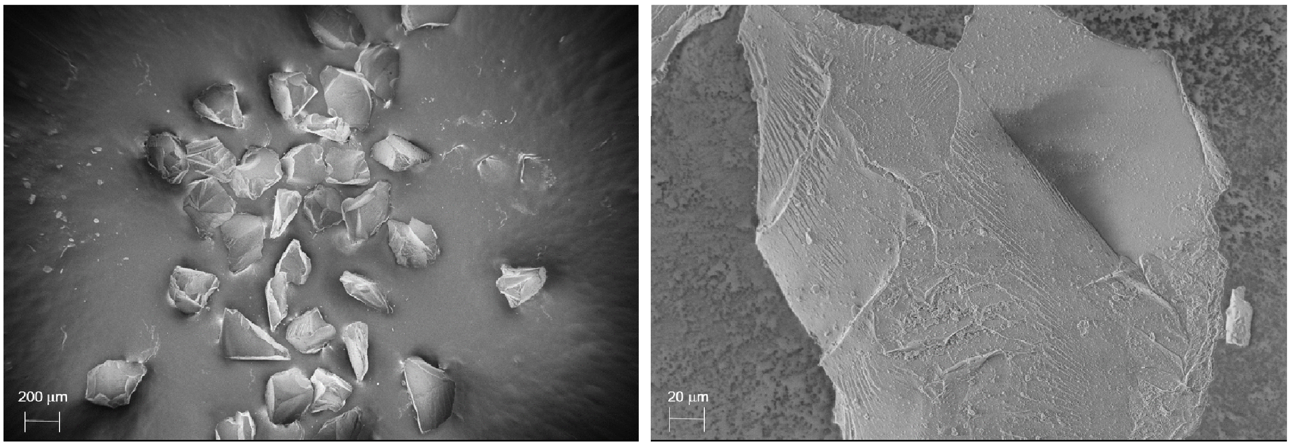

Figure S8. SEM images of bare silica nanoparticles.

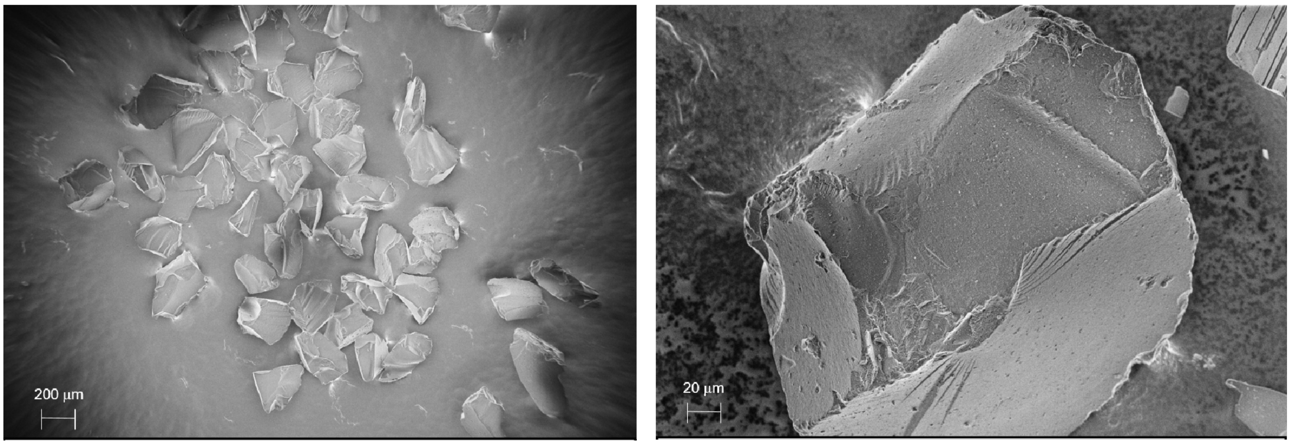

Figure S9. SEM images of QxCav functionalized silica nanoparticles.

5 Preconcentrator testing conditions

The details of the analysis cycle implemented by the mini-GC system are reported elsewhere [1], while the temperature gradients operated on the two MEMS devices are shown in Figure 3a, which reports the read-out of the actual MEMS temperature during a typical measurement cycle.

At t = 60 s the injection starts, and the pre-concentrator MEMS is rapidly heated to the injection temperature of 110°C, while the GC column is in isothermal conditions at 60 °C. At t = 90 s the temperature ramp on the MEMS GC column starts, going from 60 °C to 180°C in 150 s (at 48 °C/min). The GC column temperature dwells at 180 °C until the end of the 10 min analysis cycle.

The pre-concentrator goes off-line from the GC column at t = 100 s, after 40 s of injection, but it is kept at 110 °C until t = 210 s for cleaning purposes, reducing carry-over effects between subsequent measurement cycles. After t = 210 s and until the end of the cycle the pre-concentrator is adsorbing the sample which will be injected in the successive cycle.

6 References

[1] Condorelli, G. G.; Motta, A.; Favazza, M.; Fragalà, I. L.; Busi, M.; Menozzi, E.; Dalcanale, E.; Cristofolini, L. Grafting Cavitands on the Si(100) Surface. *Langmuir* 2006, *22*, 11126-11133.

[2] Bianchi, F.; Pinalli, R.; Ugozzoli, F.; Spera, S.; Careri, M.; Dalcanale, E. Cavitands as superior sorbents for benzene detection at trace level. *New J. Chem.* 2003, *27*, 502–509.

[3] Bianchi, F.; Mattarozzi, M.; Betti, P.; Bisceglie, F.; Careri, M.; Mangia, A.; Sidisky, L.; Ongarato, S.; Dalcanale, E. Innovative cavitand-based sol-gel coatings for the environmental monitoring of benzene and chlorobenzenes via solid-phase microextraction. *Anal. Chem.* 2008, *80*, 6423–6430.

[4] Zampolli, S.; Elmi, I.; Mancarella, F.; Betti, P.; Dalcanale, E.; Cardinali, G. C.; Severi, M. Real-time monitoring of sub-ppb concentrations of aromatic volatiles with a MEMS-enabled miniaturized gas-chromatograph. *Sens. Actuators B* 2009, *141*, 322-328.
